# Supplementary material for: Small burrowing amphipods cause major damage in a large kelp
Source: Proc Biol Sci. 2020 Apr 29;287(1926):20200330. doi: 10.1098/rspb.2020.0330 (PMC7282921; doi:10.1098/rspb.2020.0330)
Supplement: Supplementary material [file rspb20200330supp1.docx]

**Small burrowing amphipods cause major damage in a large kelp**

**– Supplementary material –**

Lars Gutow^1*^, Alistair G.B. Poore^2^, Manuel A. Díaz Poblete^3^, Vieia Villalobos^3^, Martin Thiel^3,4,5^

^1^Alfred Wegener Institute Helmholtz Centre for Polar and Marine Research, Department of Functional Ecology, 27570 Bremerhaven, Germany

^2^Evolution & Ecology Research Centre, School of Biological, Earth and Environmental Sciences, University of New South Wales, Sydney, NSW 2052, Australia

^3^Facultad de Ciencias del Mar, Universidad Católica del Norte, Coquimbo, Chile

^4^Millennium Nucleus Ecology and Sustainable Management of Oceanic Island (ESMOI), Coquimbo, Chile

^5^Centro de Estudios Avanzados en Zonas Áridas (CEAZA), Coquimbo, Chile

*Corresponding author: [lars.gutow@awi.de](mailto:lars.gutow@awi.de)

DOI: 10.1098/rspb.2020.0330

Figure S1: Morphological stages (Stages 1-4) of amphipod domiciles within stipes of subtidal individuals of *Lessonia berteroana* from Playa Blanca.

Figure S2: Typical thallus of a subtidal individual of *Lessonia berteroana* from Playa Blanca. Internode levels are numbered in sequence starting at 1 directly above the holdfast. The dashed sections of the stipe schematically illustrates the reconstruction of an undamaged stipe section. Scale = 50 cm

Figure S3: Number of amphipod domiciles on stipes of *Lessonia berteroana* from ten different sites within the kelp forest of Playa Blanca (N = 8-10)

Figure S4: Relationship between stipe length of subtidal individuals of *Lessonia berteroana* and number of amphipod domiciles in the kelp forest of Playa Blanca (N = 98)

Table S1: Average (± SD) internode propagation rate and internode biomass of subtidal individuals of *Lessonia berteroana* from Playa Blanca

Table S2: Results of the generalized linear model (GLM) to compare the average number of amphipod burrows among domiciles of different stage of infestation and among the two sampling years. The burrows were identified on stipes of subtidal individuals of *Lessonia berteroana* from Playa Blanca collected in 2011 and 2014.

Table S3: Results of the generalized linear model (GLM) to compare the average number of individuals of amphipods among the two sampling years, among domiciles of different stage of infestation, and among the two amphipod species. Amphipods of the species *Sunamphitoe lessoniophila* and *Bircenna* sp. were extracted from domiciles in stipes of subtidal individuals of *Lessonia berteroana* from Playa Blanca collected in the years 2011 and 2014.
